# Effect of Ti6Al4V Alloy Surface and Porosity on Bone Osseointegration: In Vivo Pilot Study in Rabbits

**DOI:** 10.3390/ma18092141

**Published:** 2025-05-06

**Authors:** Amparo Vanaclocha, Vicente Vanaclocha, Carlos M. Atienza, Pablo Jordá-Gómez, Víctor Primo-Capella, Jose R. Blasco, Luis Portolés, Nieves Saiz-Sapena, Leyre Vanaclocha

**Affiliations:** 1 Biomechanics Institute of Valencia, Polytechnic University of Valencia, 46022 Valencia, Spain; amparovanaclocha@hotmail.com (A.V.); carlos.atienza@ibv.org (C.M.A.); vjprimo@ibv.org (V.P.-C.); 2 Department of Surgery, Division of Neurosurgery, University of Valencia, 46010 Valencia, Spain; 3 Hospital General Universitario de Castellón, 12004 Castellón de la Plana, Spain; jorda.gomez.pablo@gmail.com; 4 AIDIMME—Metal Processing, Furniture, Wood and Packaging Technology Institute, Parque Tecnológico, Avda. Leonardo Da Vinci 38, 46980 Paterna, Spain; 5 Hospital General Universitario de Valencia, 46014 Valencia, Spain; nssapena@hotmail.com; 6 Medius Klinik, Ostfildern-Ruit Klinik für Urologie, Hedelfinger Strasse 166, 73760 Ostfildern, Germany; leyrevanaclocha@hotmail.com

**Keywords:** bone–implant interactions, osseointegration, bone–implant interface, implant surface design, titanium implants, porous implants

## Abstract

Unmodified Ti6Al4V can osseointegrate, but sometimes this capacity needs to be improved. This study aimed to see how much porosity improves osseointegration in a Ti6Al4V implant. Three types of Ti6Al4V cylindrical-shaped implants (13.00 mm length × 5.00 mm diameter) were evaluated: solid sandblasted acid-etched, sintered, and porous 3D-printed (681.00 µm average pore size). Fifteen 20-week-old nullipara female parasite-free New Zealand California white rabbits were used, employing the femoral condyle defect model and undertaking µ-CT analysis and pull-out testing eight weeks later. On µ-CT densitometric analysis, the solid sandblasted rod showed the highest new bone growth around the implant. Bone growth was higher inside the implants for the porous 3D-printed (54.00 ± 5.00 mm^3^) than for the sintered (1.00 ± 0.05 mm^3^) and zero for the sandblasted implants. In the pull-out test, there were no statistically significant differences in the ANOVA analysis between the sintered (900.00 N ± 310.00 N) and porous 3D-printed (700.00 N ± 220.00 N) implants. Such differences did exist between the sandblasted material (220.00 N ± 50.00 N) and the two other materials (sintered *p* 0.002, porous *p* 0.034). The porous 3D-printed and sintered implant pull-out strength were significantly better than that of the solid rod sandblasted implant. Still, there were no statistically significant differences between the first two.

## 1. Introduction

Patients increasingly demand higher quality, functionality, and safety levels in surgical implants without compromising biocompatibility. Additive manufacturing technologies open new doors to further improvement.

Orthopedics, Dentistry, and even Cardiothoracics widely use titanium and its alloys as a prosthetic material because of its mechanical properties [1,2], Young’s modulus [3], induction of a minimal body reaction [4], biocompatibility [5], high resistance to corrosion [6], low tissue responsiveness [7], optimal strength-to-weight ratio [8], and non-magnetic properties [9]. The most widely used alloy, Ti4Al6V, has a Young’s modulus (~110 GPa) much higher than that of human cortical bone (10–40 GPa) [10]. This mismatch can facilitate stress shielding [11,12], leading to bone atrophy with long-term implant loosening [13]. A second issue is the relative toxicity of the aluminum and vanadium ions released from the implant, which can lead to local and systemic toxicity [14,15,16,17]. Furthermore, it has limited elasticity [18], which can cause osteolysis around the implant [19]. A final drawback is its poor adhesion at the bone–implant interface [20,21]. That is why the search for new titanium alloys that best suit the ideal requirements continues. Among the improvements is the substitution of aluminum and vanadium for other ions contained in the human body, like manganese or iron [18]. Adding niobium, zirconium, or molybdenum can increase elasticity [18,22], and tantalum can improve resistance to corrosion [18]. This latter issue is critical for implants destined for the oral cavity.

Titanium’s strong biocompatibility with bacteria [23] correlates with frequent infections, particularly in oral implants [24]. This drawback is overcome by applying coverings with silver [25,26,27], zinc [28,29,30,31], magnesium [29,30,32], strontium [31,33,34], iodine [35], iron [29], doxycycline [36], or H-halamine [23]. The latest trend, though, has been to incorporate copper or zinc into the metal alloy, as those metals are tolerated in the human body but have strong antibacterial action [37,38]. This property is a definitive advancement for dental implants as they will be placed in a cavity with a huge bacterial load.

In implants, the immediate pull-out resistance relies on passive press-fit [39] and its design [40]. However, long-term implant retention depends on the invasion of its crevices by nearby bone [41] and on chemical bonding with the nearby osseous tissue [42]. The bone–implant interlocking strength is initially weak and takes a long time to develop [43].

Titanium’s osseointegration capacity is enhanced further with other coatings, like hydroxyapatite [44], lactoferrin [26], boron-containing nanostructures with calcium silicate [34,45], carbon nanoforms [46], graphite [47], phosphorus [48], chitosan [49], calcium phosphate [50,51], calcium phytate [52], calcium fructoborate [53], calcium titanate [54], tribochemical silica [34,55], polycrystalline diamond [56,57], magnesium [32] strontium ranelate [31,34], carbide [58], BMP-7 [57], and cell-adhesive tripeptides [59], among others. However, the thermal expansion mismatch between the different materials [60] often causes partial coating delamination from the titanium [61] and microcracks [62].

Another way to increase osseointegration is by administering deferoxamine [63], alfa-tocoferol [64], bisphosphonates [65], silymarin [66], tibolone [67], alendronate [68], zoledronate [69], or simvastatin [70]. However, this method is predominantly used in laboratory research with limited clinical application [31].

As surface nanotopography increases osseointegration [45,71], others create these irregular surfaces with micro-arc oxidation [72], electron beam melting [73], or direct metal laser sintering [3]. In recent years, nanoscale morphologies have attempted to improve osseointegration further [70,71,74]. The logical query is whether sintering the metal implants is enough or if a lattice structure is needed to foster better long-term osseointegration and secondary retention of the implant [75,76]. For manufacturing companies, sintering is less costly than porous structures, which is a pertinent issue in a time of cost containment [77].

Lattice structure has been applied to other materials like tantalum, finding that it also improves osseointegration and long-term secondary implant retention [78,79]. However, the high melting point of tantalum (2996 °C) [80] makes its management awkward, contrary to titanium (1668 °C) [81], which is easier to forge [82].

An advantage of porous Ti6Al4V alloy structures is that they have an elastic modulus closer to that of the cortical human bone than the solid rod ones, minimizing the elastic mismatch and, thus, the risk of stress shielding [7,83,84]. This consideration is crucial when designing any new implant [85]. Additionally, the thickness of the titanium walls created with 3D technology is about 150 µm, which is very close to one of the human bone trabeculae (100–140 µm) [86]. Today, the currently available technology allows the creation of lattice implants with characteristics close to bone [87].

Porous implants allow the colonization of neovascularization (new blood capillaries) and bone cells (migration and adhesion of osteoblastic and osteoclast cells) [88]. However, to achieve effective bone ingrowth, implants require pores of 600–800 μm, as the pores range from 100 to 150 μm in the cortical and 500 to 600 μm in the cancellous bones [84,89,90]. The optimal size for the strongest osseointegration seems to be 626 µm [91]. This pore size also improves the mechanical properties of the implant, making them closer to those of the bone, particularly the elastic modulus and compressive strength [92]. Additionally, the fatigue stress decreases as the porosity increases [93].

Bone growth inside a porous Ti6Al4V implant compared to an acid-etched solid rod or a sintered one has been studied in a rabbit midshaft tibial model and tested with Core Beam Computed Tomography (CBCT), Micro Computed Tomography (µCT), and histological studies [84,94,95]. Unfortunately, the increase in secondary retention capacity provided by this bone grown inside the implant was not studied [84,94,95,96].

Our study compared in vivo the osseointegration capacity of three types of titanium alloy (Ti6Al4V) cylindrical implants: a solid sandblasted acid-etched rod, a solid sintered rod, and a porous implant with pore dimensions between 600 and 800 μm. The primary objective was to see if this porous titanium alloy structure allowed bone growth inside it and how big this bone invasion was. Our second objective was to see how much this bone growth inside the lattice structure improved the implant anchoring to the nearby bone. Our third objective was to compare the secondary retention capacity of the three types of implants, namely the solid, the sintered, and the lattice ones, in vivo in rabbits at eight weeks.

## 2. Materials and Methods

### 2.1. Ti6Al4V Implant Designs

**Figure 1:**
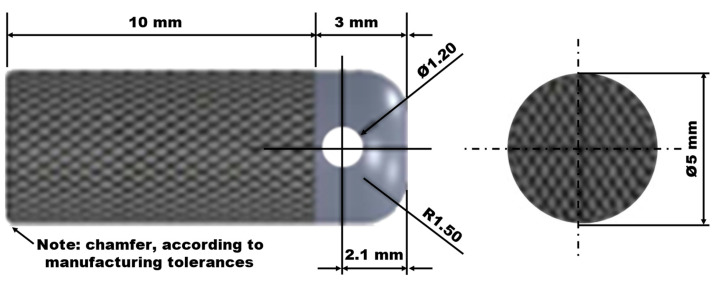
Implant geometry and dimensions.

Three types of Ti6Al4V alloy implants were designed, all with the same cylindrical shape and dimensions (length including its crown 13.00 mm and diameter 5.00 mm) (Figure 1). As the implants had no turns, the surface type and lattice structure were the only variables in this study. In addition, each implant had a hole in its crown to manage during manufacturing, surgical implantation, and pull-out tests, while the striped surface was the area that differed for each implant type.

**Figure 2:**
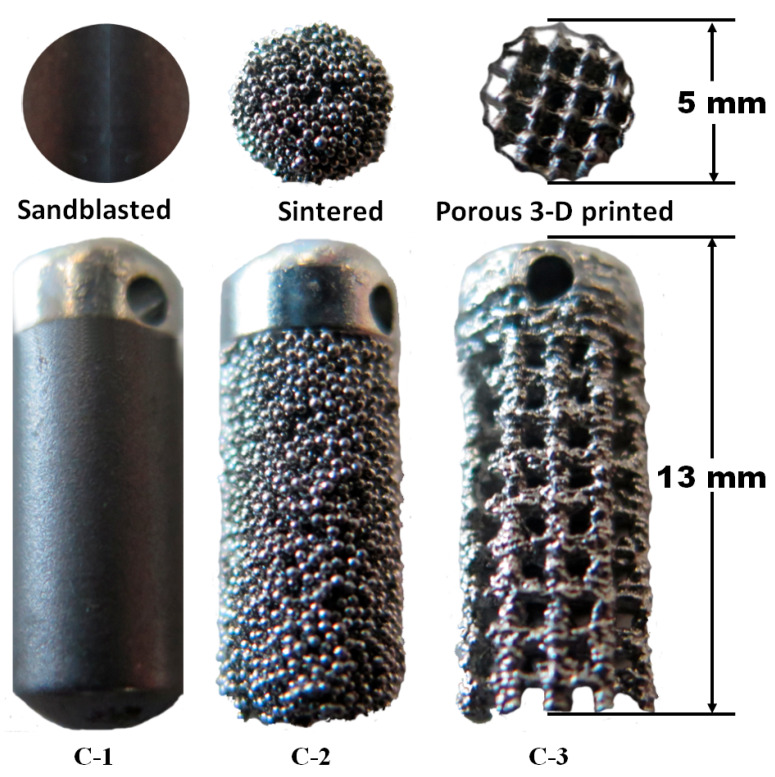
The three types of Ti6Al4V alloy implants evaluated in this study.

Each implant type had a different manufacturing technique (Figure 2). The first (C-1) was a sandblasted acid-etched solid rod. The second (C-2) was a solid rod sintered with 0.50 mm titanium microspheres and a 260.00 µm average pore diameter. The third (C-3) was a 3D-printed porous Ti6Al4V with an average pore diameter of 681.00 µm.

The C-1 solid rod underwent sandblasting with corundum grit (Al_2_O_3_, 50.00–75.00 µm in diameter, five minutes at 0.80 MPa pressure), cleaned ultrasonically with acetone, ethanol, and distilled water and acid-etched (0.50 M H_2_SO_4_) (Eckermann Dental Implant System, Almoradí, Alicante, Spain).

C-2 was a solid rod placed in a furnace at 1200 °C, at a constant speed, and in a controlled atmosphere [97] to apply a 1.20 mm thick layer of sintered titanium spheres (Bio-vac SA, Paterna, Valencia, Spain).

C-3 was a porous implant manufactured from Ti6Al4V powder with the technology powder bed fusion using an electron beam (PBF-EB/M) (Arcam A2—GE Additive, Gothenburg, Sweden) with a 681.00 μm average pore size. This equipment used a high-power electron gun to heat powdered metal and, layer by layer, print it. Then, the implants were washed for 48 h in an ethanol/water mixture (80%/20%), leaving them for 72 h in 100% ethanol before drying them with air. The porosity of this implant was 49.7 ± 1.17%, the Young’s modulus 92.54 ± 7.12 GPa, and the elastic modulus 2615.0 ± 17.12 MPa.

After manufacturing, each implant was washed, packed, and sterilized with 2.5 Mrad gamma (Gamma cell 220 Co irradiating Unit; IONISOS Ibérica, Tarancón, Spain).

### 2.2. In Vivo Ti6Al4V Alloy Implant Study

The in vivo experimental design, surgical procedure, and analysis of the data followed UNE-EN ISO 10993-6:2007 [98], UNE-ISO 10993-11:2006 [99], and ASTM F 763-04 [100]. The research was undertaken according to the Dirección General de Producción Agraria de la Consellería de Agricultura, Pesca y Alimentación, as well as the Spanish [101] and Comunitat Valenciana [102] animal research laws. The Polytechnic University of Valencia Ethical Committee, Spain, approved this study (INIDIV/2009/072).

This study was undertaken in rabbits using the femoral condyle defect model [47]. Fifteen twenty-week-old nullipara female parasite-free New Zealand California white rabbits were used, weighing 3.65–3.90 kg, born and raised in the Instituto de Biomecánica de Valencia. This animal’s rapid bone growth made it suitable for our studies [103]. To be eligible, animals should be healthy, have no physical defects at all, and should not have been used in any other research study. They were weighed once a week during our study.

Osteoporosis of the research animals was ruled out with preoperative plain X-ray studies and Dual Energy X-ray Absorptiometry (DEXA) scans (Norland, Fort Atkinson, WI, USA). Only rabbits with a +1 result were used.

A veterinarian surveilled the animals during the whole study.

Each animal was weighed before the surgical procedure to calculate the doses required for each drug. First, Xylazine 2% (2.50 mg/kg), Ketamine hydrochloride (17.50 mg/kg), and Enrofloxacin (antibiotic, 3.5 mg/kg) were administered intramuscularly. Then, the animals were anesthetized with Propofol 1%, 3.00 mg/kg, administered intravenously in a bolus injection in a marginal vein of the ear, followed by a continuous infusion of 21.00 mg/kg/h. After placing the animal supine, both legs were shaved with an electrical clipper, scrubbed with povidone–iodine, and the surgical field sprayed with an 80% ethanol povidone–iodine mixture.

**Figure 3:**
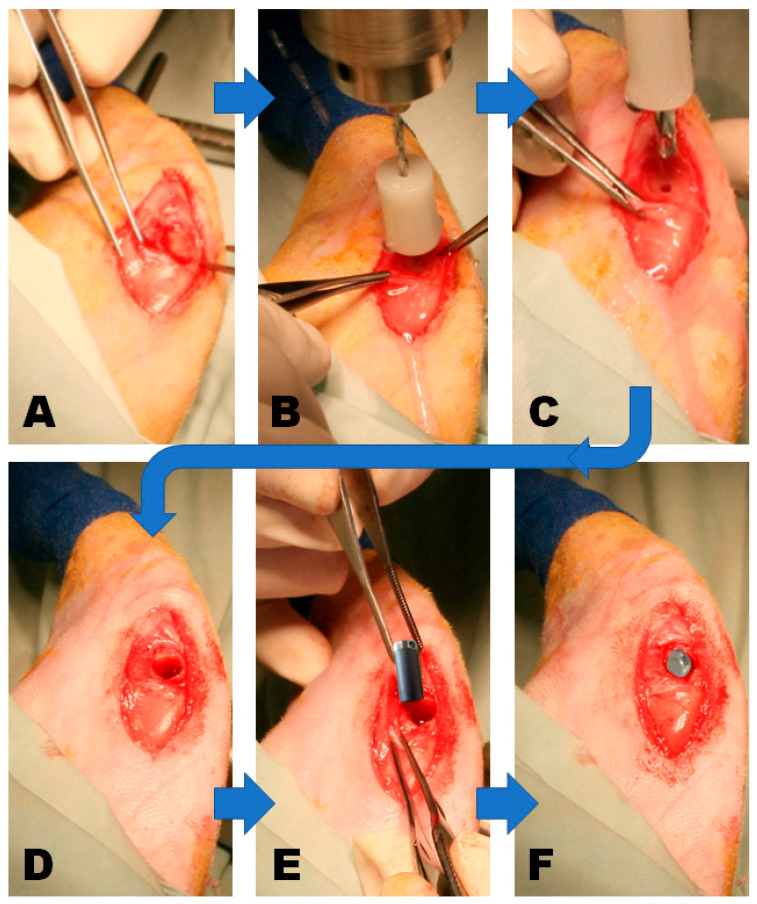
Surgical steps to implant the selected Ti6Al4V alloy in the medial rabbit’s knee condyle with its crown outside the bone to allow the pull-out test. Note that the implant shown in Figure 3E has been edited to illustrate the actual shape of the implant better. (**A**) surgical incision at the rabbit femoral condile; (**B**) first dril to insert the implant; (**C**) second dril to enlarge the space for the implant; (**D**) drill hole ready to accept the implant; (**E**), implant ready to be implanted; (**F**) implant already in place with its crown coming out of the bone to allow the pull-out tests.

Under aseptic conditions, an incision was made on the medial side of the knee on the projection of the medial femoral condyle (Figure 3A). A cylindrical defect was generated with dental drills of increasing diameters of 2.00, 3.50, and 5.00 mm and a depth of 10.00 mm. At all times, the drill was held perpendicular to the longitudinal axis of the femur, and the hole reamed gently (Figure 3). During the drilling, a physiological saline solution was used to avoid overheating of the nearby bone, as local heating over 47 °C impairs bone healing [104].

Next, the cavity was thoroughly rinsed with a lukewarm physiological saline solution to remove any bone debris (Figure 3D). Then, each titanium implant was centered in the drill hole and inserted press-fitting (Figure 3E), leaving its crown outside the nearby bone to allow free access to its hole when performing the pull-out tests (Figure 3F). Finally, the position of each implant was confirmed with plain X-ray images (Genoray Zen 7000, Gyeonggi-do, Republic of Korea).

The wound was inspected, hemostasis was achieved, and sutured layer by layer with resorbable 2-0 Vycril sutures. Then, the procedure was repeated on the other leg of each rabbit, implanting different implant models on each side. Ten legs received the C-1, C-2, and C-3 implants, respectively.

The same surgeon performed all the surgical procedures.

For the first three postoperative days, animals received Meglumine (anti-inflammatory, 1.00 mg/kg/day, subcutaneously), Butorfanol (analgesic, 0.40 mg/kg/day, intramuscularly), and Enrofloxacin (antibiotic, 0.40 mL/kg/day, intramuscularly).

After the surgical procedure, each animal was housed in a separate cage, with a 12 h day/night cycle in an air-conditioned room, free movement inside their cells, and ad libitum access to food and water.

The skin of the rabbits was inspected daily for the first ten days, looking for any signs of infection or abnormal behavior. Animals were included in the study if, in the eight-week follow-up, there were no signs of persistent soft tissue edema, abnormal inflammatory response, bone deformation, fractures of the condyle of the femur, or adverse reactions to the implants. Should that be the case, the rabbit was replaced by a new one with the same type of implants that the previous one had. Fortunately, no animal died during the surgery or postoperative periods.

**Figure 4:**
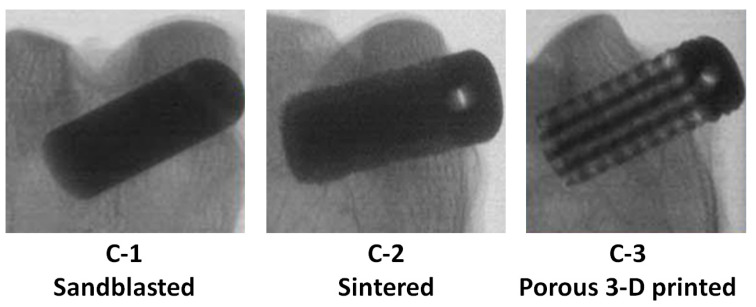
Implanted sample plain X-ray image. On the left image is C-1, the center C-2, and on the right side, C-3.

Eight weeks later, the rabbits were euthanized with an intravenous sodium pentobarbital overdose (100.00 mg/kg) administered in a single bolus through an ear vein. The condyles of the rabbits were X-rayed to confirm the presence of the implant and rule out osteolysis around them (Figure 4), as that would indicate implant loosening. In that event, animals were excluded from the study.

Under sterile conditions and careful soft tissue dissection, the femoral condyles were removed with the implants still in place and stored at −80 °C until testing. Other research groups also follow this method of conservation as it does not seem to modify the results significantly [105,106]. Then, the tissues surrounding the implantation zone were assessed microscopically, looking for any adverse reactions.

### 2.3. Evaluation of Ti6Al4V Implant Osseointegration

It was evaluated with a µ-CT densitometric analysis (Skyscan1176; Skyscan, Kontich, Belgium) and a pull-out test. The tests had to be performed in this order because the latter altered irreversibly the bone–implant interface.

### 2.4. Densitometric Analysis with µ-CT and 3D Reconstruction

**Figure 5:**
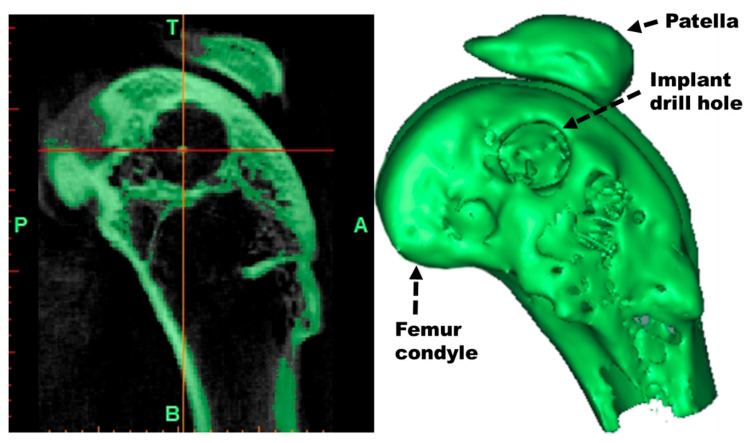
µ-CT image (**left**) and 3D reconstruction (**right**).

The µ-CT images were provided in DICOM format (DCM) and were processed with the MIMICS program (Materialize BV, Leuven, Belgium), creating a 3D reconstruction (Figure 5). Finally, the volume and average density of regenerated bone were analyzed by isodensity and callus formation (NETEOUS, Biomechanics Institute of Valencia, Valencia, Spain and INGECOT, University of Oviedo, Oviedo, Asturias, Spain).

The µ-CT scanning parameters were as follows: source voltage 100 kV, current source 100 μA, exposure time 1900 ms, image pixel size 17.55 μm, Al + Cu filter, 0.40° (360°) rotation step (deg), frame averaging three, and random movement twenty-five. In addition, the reconstruction parameters were smoothing = 1, ring artifact correction = 15, and beam and hardening correction (%) = 20.

The µ-CT study variables were the Bone Volumetric Fraction (BV/TV), Bone-Specific Surface (BS/BV), Bone Surface Density (BS/TV), Trabecular Pattern Factor (Tb.Pf), Degree of Anisotropy (DA), Intersection Surface (i.S), volumetric Bone Mineral Density (vBMD), and bone growth around the implant and inside it.

Bone Volumetric Fraction (BV/TV). In the Region of Interest (ROI), Bone Volume (BV) is the space occupied by bone, and Total Volume (TV) is the entire bone volume plus the “empty” spaces. The relationship between BV and TV is known as the bone volumetric fraction (BV/TV) and describes the percentage occupied by bone.

Bone-Specific Surface (BS/BV) and Bone Surface Density (BS/TV). The Bone-Specific Surface BS/BV is the ratio between the Bone Surface (BS) and the Bone Volume (BV), 3D-measured within the interest volume. Therefore, it is a valuable parameter to characterize the trabeculae thickness and complexity. The BS/TV ratio, known as the Bone Surface Density, is the ratio between the bone surface and the total volume analyzed.

The Trabecular Pattern Factor (Tb.Pf) is the relative convexity or concavity index of the bone surface. The trabecular surface concavity implies connectivity and surface convexity, which are disconnected and isolated structures. The higher its value, the less connected the bone trabeculae are.

The Degree of Anisotropy (DA) measures the object’s symmetry and the presence or absence of structures aligned in a specific direction. Its values range from 0 to 1, where 0 is full isotropy and 1 is full anisotropy.

The intersection surface (i.S.) is the contact surface between the implant and bone. It measures the ROI contact surface of the bone at any distance from the surface of the implant, providing an additional dimension of its depth. The i.S profile is the percentage of surface in contact with bone concerning the total surface (i.S/TS).

The volumetric bone mineral density (vBMD) was calculated by scanning two calcium hydroxyapatite (CaHA) phantoms of known densities (250.00 and 750.00 mg/cm^3^) under the same conditions used for the analysis of the bone. Then, the samples were scanned, and the vBMD was calculated by directly calibrating against the attenuation coefficients of the phantoms.

The bone growth around the implant was calculated by analyzing different distances from it (1.00 → 0.0703–0.4216 mm, 2.00 → 0.4216–0.7729 mm, 3.00 → 0.7729–1.1242 mm, 4.00 → 1.1242–1.4755 mm and 5.00 → 1.4755–1.8269 mm) with a conversion of 35.1321 μm/pixel, discarding the first two pixels to overcome the distortion induced by the Ti6Al4V alloy.

Finally, the volume occupied in the ROI by the implant and the bone was calculated, as well as the one not occupied by one or the other. Then, the amount of bone inside the implant was also calculated. Finally, the percentage of bone occupied by bone was obtained over the total volume it could occupy.

### 2.5. Pull-Out Test

Once the µCT studies were finished, the pull-out test was conducted to determine the static resistance to the extraction of the implant from the condyles of the rabbits. This test was performed with the INSTRON 8874/287 (Canton, MA, USA) with a 25,000 N load cell.

**Figure 6:**
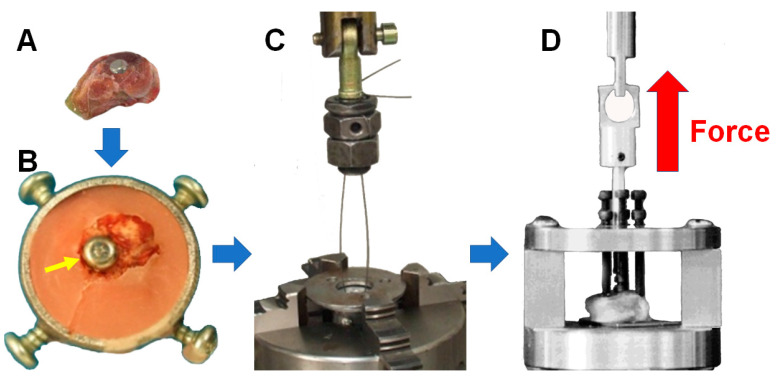
Pull-out test assembly: (**A**) thawed rabbit’s femoral condyle without soft tissues but with the Ti6Al4V implant in place; (**B**) femoral condyle embedded in acrylic bone cement containing the implant (depicted by a yellow arrow); (**C**) steel cable passed through the implant’s crown hole and (**D**) final arrangement before starting the pull-out test.

In preparation for this test, the condyles of the rabbits were thawed for 4–5 h at room temperature (Figure 6A). Subsequently, they were cemented with acrylic bone cement (SR Triplex Cold, Ivoclar Vivadent AG, FL-9494 Schaan; Liechtenstein) in plastic bowls. The medullary canal was covered to prevent bone cement from entering inside it, as that might enhance the resistance of the implant to being pulled out, distorting the results (Figure 6B). Next, the assembly was held with clamps in the lower jaw of the testing machine to guarantee stability throughout the test. It was aligned with the axis of the application of the force. Finally, a steel cable was passed through the hole in the crown of the implant and held to the upper clamp of the testing machine (Figure 6C).

Once everything was ready, force was exerted to extract the implant from the bone at 0.05 mm/s, scroll control under 23 °C, and 46% humidity (Figure 6D). The specimens were sprayed with a lukewarm 0.9% sodium chloride solution every five minutes to prevent unwanted desiccation. The trial ended with implant removal or assembly failure, recording the maximum force required for each. The pull-out force–displacement curves, maximum extraction force, and tangential stiffness were calculated.

## 3. Statistical Analysis

It was performed with MS Excel (Microsoft Corporation, Redmond, WA, USA) and the free statistical analysis software R (R Development Core Team, R version 4.4.3, R Foundation for Statistical Computing, Vienna, Austria, https://www.r-project.org/, accessed on 14 January 2024). First, the Student *t*-test was performed to find whether statistically significant differences existed between the three implant types. With the results of the pull-out tests, a statistical analysis was conducted to look for any statistically significant differences in the extraction force with ANOVA tests. A *p*-value < 0.05 was considered statistically significant. The sample size was determined based on the research team’s experience with animals, seeking the minimum possible number of rabbits that would ensure comparable results and, at the same time, avoid unnecessarily sacrificing more animals. The research team decided to analyze 10 implants of each of the three implant types. Therefore, fifteen rabbits were required as both legs of each animal were implanted.

## 4. Results

None of the animals were lost due to infection or trauma to the operated site. No implant had to be discarded due to loosening, mobility, or loss. No inflammatory reaction or rejection of the materials was seen in any of the rabbits.

### 4.1. Bone Growth AROUND the Implant

The solid rod sandblasted acid-etched C-1 implants showed the most significant new volume of bone growth around the implant at all distances. The bone could grow only around this type of implant as it was a solid, non-porous material. The mean surface BMD was 438.49 mg/cm^3^ for C-1, 382.99 mg/cm^3^ for C-2, and 393.47 mg/cm^3^ for C-3 (Table 1).

The vBMD was also higher for C-1, middle for C-2, and lower for C-3, but there were no statistically significant differences between them (Table 1).

### 4.2. Bone Growth INSIDE the Implant

The C-1 sandblasted acid-etched Ti6Al4V alloy implant could not have bone growth inside it because it was a solid metallic rod. Thus, the bone could grow only around it.

Next, the bone growth inside the sintered (C-2) and porous (C-3) implants was analyzed. The volume that the bone could occupy was much higher in C-3 than in C-2 (62.00 ± 4.00 mm^3^ versus 1.00 ± 0.04 mm^3^), and accordingly, the total volume of bone grown in the implant was also much higher for C-3 (54.00 ± 5.00) than for C-2 (1.00 ± 0.05 mm^3^) (Table 2). C-3 provides more space for new bone growth inside the implant than C-2, and this bone inside the implant should improve implant retention in the long term.

**Figure 7:**
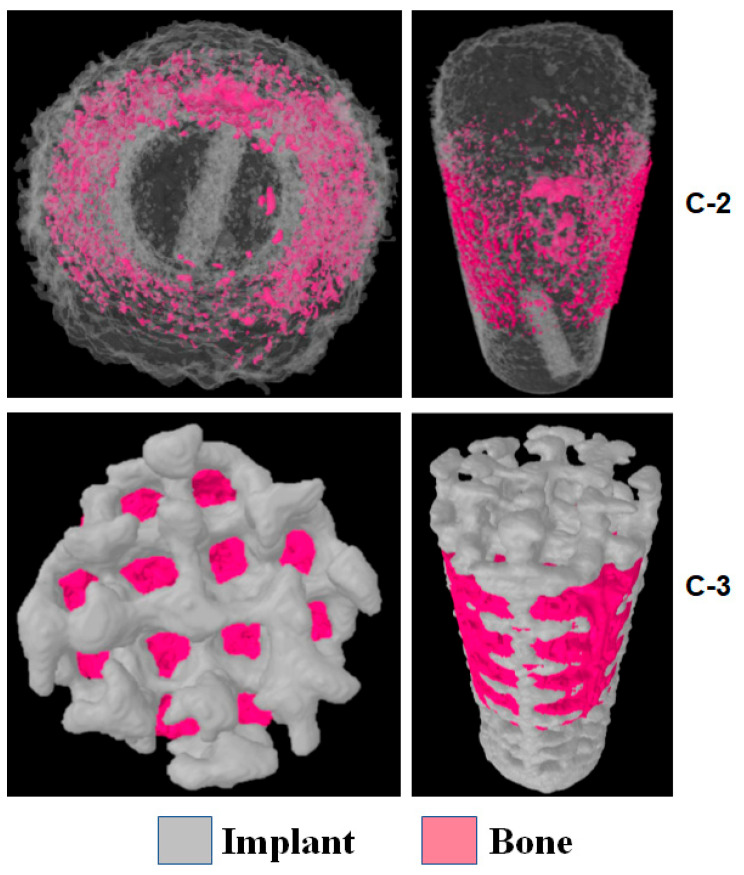
Bone growth inside C-2 and C-3 implants.

Figure 7 shows the 3D images of the bone growth inside the C-2 and C-3 implants, depicting that it was much higher for the latter. It must be considered that this bone inside the implant should increase long-term osseointegration. Once this new bone matures over time, it might also increase the capacity of secondary retention.

### 4.3. Pull-Out Test Results

In the ANOVA test, there were no statistically significant differences in the extraction forces between C-2 (900.00 N ± 310.00 N) and C-3 (700.00 N ± 220.00 N). Contrarily, these differences existed between C-1 (220.00 N ± 50.00 N) and the other two materials (*p* 0.002 for C-2) and (*p* 0.034 for C-3), respectively.

## 5. Discussion

This study shows that in the model of the femoral condyle in the rabbit, a porous lattice structure with a 681 μm average pore size shows adequate bone growth inside it. At eight weeks, this bone growth inside the porous implant correlates with an improvement in the pull-out force required to extract it. This difference is statistically significant when compared to the solid sandblasted acid-etched rod but not with the sintered one, as reported by other research groups [76]. Additionally, our study shows that sandblasting the titanium alloy implants induces a strong new bone formation around them, as seen by others [107,108].

The lattice structure with an average pore size of 681 µm has less mechanical resistance than the solid or sintered rods, but it is strong enough to support all mechanical stresses. Its Young’s modulus is lower than that of solid Ti6Al4V implants, but it is still double that of the human cortical bone.

The animal model selected is adequate to the aims of our study. The rabbit is an ideal animal for this type of research as it has a bone structure similar to that of a human but with a bone that heals twice as fast [109,110,111]. The bone growth data obtained from implanting biomaterials in this animal have been found to be transferable to humans [110,112]. This research model is adequate as it provides mechanical loading during the movements of the animal, such as walking and jumping [110,112]. Both legs in each rabbit were used to reduce the number of animals that had to be inconvenienced and sacrificed and to compare different implant types in the same rabbit [95].

The age of the animals also matters. Female rabbits are considered adults when they are older than 14 weeks and weigh more than 3 kg [113,114]. Using younger animals is not recommended as their bone growth capacity does not correlate with that of adult humans [110,111]. In the present study, only female rabbits 20 weeks old weighing 3.65–3.90 kg were used.

The time the implant remains inserted in the bone affects the push-out strength [91]. In some studies, at four or six weeks, in the model of the femoral condyle in the rabbit, there were no statistically significant differences in the push-out strength between hollow versus solid screws, which was present at twelve weeks [7,115]. Others have seen robust bone growth inside the porous implants at 13 and 20 weeks [84]. In the present study, at eight weeks, there were minimal differences in the pull-out strength of the porous implants compared to the sintered ones. Eight weeks of implant insertion in the rabbit is accepted in research [67,95]. Still, if the animals were left to survive for extended periods [84], even years, these differences might have achieved statistical significance. This fact must be taken into consideration because, in clinical practice, patients are expected to survive for years after having the implants inserted. The bone around and inside implants matures over time, increasing its secondary retention capacity [57,84,116].

Sintering improves surface osseointegration compared to solid titanium implants [105,117,118], particularly if any covering is added [46,119]. However, only porous ones allow new bone growth inside them [78], which increases the osseointegration capacity, secondary implant retention [119,120], and osseous stability [105]. The lattice structures provide a larger bone–implant interface area with better long-term bone growth [120] and avoid the debonding side-effect seen in other types of implants [51]. An additional advantage is that porous 3D-printed implants reduce the mismatch between implant and bone [121], minimizing stress shielding [115], which can lead to bone loss around the implant and its failure [122].

However, not all are advantages as the lattice structure weakens the structural strength of the porous implant [123,124], a detail of particular importance for weight-bearing applications like major joint or spinal surgeries. Some researchers have minimized this fatigue resistance by a technique known as hot isostatic pressing [120]. It has been found that implants with a porosity of 59.86% respond the best mechanically [125]. The pore shape is also crucial as honeycomb lattice structures have the highest elastic modulus, intermediate for round-shaped ones, and the lowest for square-shaped ones [126]. The present study did not show fatigue or material fracture in any of our implants, in spite of the weight-bearing requirements and with a porosity of 49.7 ± 1.17%. The shape of the pores was round-shaped. Still, the implantation time was relatively short (eight weeks) to draw definitive conclusions.

Pore size is another issue to consider, both in sintered and porous implants. Although pore sizes of 100 to 150 µm allow a weak neovascularization ingrowth and new bone formation [34,127,128], those with 600 µm provide the best bone ingrowth [91], mechanical strength, and secondary retention capacity [129]. Meanwhile, with 700 and 1500 µm, bone growth happens mainly along the metallic structure but not inside the pores [34,130]. The present study shows that in the model of the femoral condyle in the rabbit at eight weeks, the bone invaded all the spaces inside the porous Ti6Al4V alloy implant with a 681 µm pore size.

Coating with other materials is an important consideration. It makes no difference for lattice structures with pore sizes below 200 µm but helps to promote osseointegration when they exceed 400 µm [131]. In the present study, no surface covering was applied to the implants as the only variable to study was whether it was solid, sintered, or porous.

A final consideration is the liberation of ions from the Ti6Al4V alloy implant. An interesting study compared the amount of this problem, comparing milled versus porous additive-manufactured implants, and found no statistically significant differences between them [17]. We have not studied this issue in the present research.

Regarding limitations, the number of research animals is reduced, which is common in this type of study [84], and the survival time is relatively short. It would be interesting to know if prolonging survival improves bone maturation inside the implant and pull-out strength. Although implants were subjected to weight-bearing, compression and shearing forces were not analyzed. Different implant morphologies or shapes were not studied, and this would undoubtedly alter the results. Additionally, histomorphological studies were not performed. Our group used them in past studies and found that the µ-CT is just as good as that reported by other research groups [132,133,134].

Additionally, the pull-out or push-out test cannot be performed if the plan is to perform the histomorphometry afterward. This decision was a compromise on our side because we had to use double the number of animals, half of them for the µ-CT and pull-out test and half of them for the µ-CT and histomorphometry studies. This fact had double the economic costs and the number of rabbits to be inconvenienced and sacrificed.

Finally, the Young’s modulus of the C-3 porous implant is lower than that of the T16Al4V alloy but still double that of the human cortical bone.

The strengths of the present study are that it has three groups, and the allocation of implants was random in the different animals and the sides of each rabbit. The µ-CT test provides solid data to analyze the new bone formation and its location in the three different Ti6Al4V implants. Few studies have reported performing the push-out test [47,135], and we have not found any other research that used the pull-out test. However, both are considered reliable methods to foster implant osseointegration, increasing secondary retention capacity.

## 6. Conclusions

Sandblasting titanium alloy implants induces a strong new bone formation around them.

The lattice structure with an average pore size of 681 µm has less mechanical resistance than the solid or sintered rods, but it is strong enough to support all mechanical stresses.

The porous implant manufactured by PBF-EB/M technology allows the bone to grow inside it.

The pull-out strength of the porous and sintered implants was 75% and 70% higher than that of the solid acid-etched rod. The porous one had a pull-out strength 22% greater than the sintered one, but these differences were not statistically significant.

## Figures and Tables

**Table 1:** New bone formation around each type of implant.

Pixels	Distance (mm)	IMPLANT TYPE	BV/TV (%)	BS/BV (mm^3^)	BS/TV (mm^3^)	Tb.Pf (mm^3^)	DA	LS/TS (%)
2–12	0.0703–0.4216	C-1	63.00 ± 5.00	21.00 ± 2.00	13.00 ± 1.00	−1.50 ± 0.01	0.70 ± 0.06	69.00 ± 6.00
		C-2	42.00 ± 3.00	28.00 ± 2.00	12.00 ± 1.00	0.80 ± 0.03	0.50 ± 0.03	33.00 ± 3.00
		C-3	31.00 ± 3.00	24.00 ± 2.00	7.00 ± 1.00	7.00 ± 0.04	0.30 ± 0.01	26.00 ± 6.00
12–22	0.4216–0.7729	C-1	49.00 ± 4.00	22.00 ± 2.00	11.00 ± 1.00	4.00 ± 0.03	0.70 ± 0.05	47.00 ± 3.00
		C-2	40.00 ± 3.00	25.00 ± 2.00	10.00 ± 1.00	−0.80 ± 0.05	0.50 ± 0.03	28.00 ± 2.00
		C-3	25.00 ± 2.00	24.00 ± 2.00	6.00 ± 0.60	8.00 ± 0.07	0.40 ± 0.02	19.00 ± 2.00
22–32	0.7729–1.1242	C-1	42.00 ± 3.00	23.00 ± 2.00	10.00 ± 0.80	5.00 ± 0.37	0.70 ± 0.05	38.00 ± 2.00
		C-2	38.00 ± 3.00	24.00 ± 2.00	9.00 ± 0.80	0.10 ± 0.01	0.50 ± 0.04	27.00 ± 2.00
		C-3	24.00 ± 2.00	23.00 ± 2.00	6.00 ± 0.50	8.00 ± 0.73	0.40 ± 0.02	18.00 ± 2.00
32–42	1.1242–1.4255	C-1	37.00 ± 2.00	24.00 ± 2.00	9.00 ± 0.70	5.00 ± 0.47	0.70 ± 0.05	32.00 ± 2.00
		C-2	35.00 ± 2.00	24.00 ± 2.00	8.00 ± 0.70	1.00 ± 0.01	0.50 ± 0.03	25.00 ± 2.00
		C-3	23.00 ± 2.00	22.00 ± 2.00	5.00 ± 0.50	9.00 ± 0.72	0.40 ± 0.02	17.00 ± 1.00
42–52	1.4755–1.8269	C-1	29.00 ± 2.00	26.00 ± 2.00	8.00 ± 0.70	8.00 ± 0.67	0.70 ± 0.05	24.00 ± 2.00
		C-2	30.00 ± 3.00	24.00 ± 2.00	7.00 ± 0.60	2.00 ± 0.02	0.60 ± 0.03	21.00 ± 2.00
		C-3	21.00 ± 2.00	22.00 ± 2.00	5.00 ± 0.40	9.00 ± 0.71	0.50 ± 0.02	16.00 ± 1.00

**Table 2:** Bone growth inside C-2 and C-3 implants.

	C-2	C-3
ROI total volume (mm^3^)	110.00 ± 90	104.00 ± 8.00
Implant occupied ROI volume (mm^3^)	109.00 ± 8.00	41.00 ± 4.00
Implant occupied ROI volume %	99.00 ± 9.00	40.00 ± 3.00
Implant NOT occupied ROI volume (mm^3^)	1.00 ± 0.04	62.00 ± 4.00
Implant NOT occupied ROI volume %	1.00 ± 0.03	60.00 ± 4.00
ROI volume occupied by bone (mm^3^)	1.00 ± 0.05	54.00 ± 5.00
Bone NOT occupied ROI volume %	94.00 ± 7.00	86.00 ± 7.00

## Data Availability

The data presented in this study are available on request from the corresponding author. The data are not publicly available due to privacy.
